# Supplementary material for: Expectation maximization based framework for joint localization and parameter estimation in single particle tracking from segmented images
Source: PLoS One. 2021 May 21;16(5):e0243115. doi: 10.1371/journal.pone.0243115 (PMC8139521; doi:10.1371/journal.pone.0243115)
Supplement: S1 Text — (PDF) [file pone.0243115.s001.pdf]

S1 Text. Detailed description of U-EM.

Consider a generic dynamic state space system depending on a parameter θ

$$X_{t+1} = f_t(X_t, w_t, \theta), \tag{1a}$$

$$Y_t = h_t(X_t, v_t, \theta). \tag{1b}$$

The U-EM scheme starts with an Unscented Kalman Filter (UKF) to get the filtered estimate of the state X and then applies an Unscented Rauch-Tung-Striebel Smotther (URTSS) to return the posterior probability densities $p(X_t|Y_{1:t})$ needed for the EM algorithm. These steps are described below. Throughout, N refers to the total number of time points (number of images in the SPT application) so that $t = 1, 2, \ldots, N$.

Unscented Kalman Filter (UKF)

The goal of the UKF is to form a Gaussian approximation of the distribution of the state X (the location of the particle being tracked in the SPT data). Generically, this distribution is given by

$$p(X_t|Y_{1:t}) \simeq \mathcal{N}\left(\mathbf{m}_t, \mathbf{P}_t\right), \quad t = 1, ..., N, \tag{2}$$

where $\mathcal{N}\left(\mathbf{m}_t, \mathbf{P}_t\right)$ denotes a Gaussian (also known as a Normal) distribution of mean $\mathbf{m}_t$ and covariance $\mathbf{P}_t$. These distributions at each time step are found as follows.

1. Prediction step: First calculate the deterministic *sigma points*, $\mathcal{X}$, according to

$$\mathcal{X}_{t-1}^{(0)} = \mathbf{m}_{t-1}, \tag{3a}$$

$$\mathcal{X}_{t-1}^{(i)} = \mathbf{m}_{t-1} + \sqrt{(n+\zeta)}\left[\sqrt{\mathbf{P}_{t-1}}\right]_i, \ (i = 1, \ldots, n), \tag{3b}$$

$$\mathcal{X}_{t-1}^{(i+n)} = \mathbf{m}_{t-1} - \sqrt{(n+\zeta)}\left[\sqrt{\mathbf{P}_{t-1}}\right]_i, \ (i = 1, \ldots, n), \tag{3c}$$

where n is the dimension of the state, $\mathbf{m}_0$ and $\mathbf{P}_0$ are randomly initialized, $[\cdot]_i$ denotes the ith column of the matrix, $\sqrt{A}$ is the matrix square root of A, and ζ is a scaling parameter defined by $\zeta = \alpha^2(n+\kappa) - n$. The parameters α and κ are determined by the user to tune the algorithm performance. The parameter α determines the spread of the sigma points around the mean and is usually taken

in the interval $(0,1]$, while κ is a secondary scaling parameter which is usually set to $3-n$ (see [22] for details). While algorithms for tuning these parameters have been developed[1], in our experience based on extensive simulations, selecting α =1 returns good performance across a range of diffusion coefficients. As seen in Case 3 of the simulation results, at large diffusion coefficients the algorithm fails independent of the parameter tuning.

The sigma points are then propagated through the motion model Eq (1a)

$$\hat{\mathcal{X}}_t^{(i)} = f(\mathcal{X}_{t-1}^{(i)}, w_{t-1}, \theta),\ i = 0, ..., 2n, \tag{4}$$

and combined to produce the predicted mean and covariance at time t given data up to time $t-1$ according to

$$\mathbf{m}_t^- = \sum_{i=0}^{2n} W_i^{(m)} \hat{\mathcal{X}}_t^i, \quad \mathbf{P}_t^- = \sum_{i=0}^{2n} W_i^{(c)} (\hat{\mathcal{X}}_t^{(i)} - \mathbf{m}_t^-)(\hat{\mathcal{X}}_t^{(i)} - \mathbf{m}_t^-)^T + Q_{t-1}, \tag{5}$$

where Q_{t-1} is the covariance matrix of the process noise w_t in Eq (1a). The weights in Eq (5) are given by

$$W_0^{(m)} = \frac{\zeta}{n+\zeta}, \tag{6a}$$

$$W_0^{(c)} = \frac{\zeta}{n+\zeta} + (1 - \alpha^2 + \beta),\ i = 1, \ldots, 2n, \tag{6b}$$

$$W_i^{(m)} = W_i^{(c)} = \frac{1}{2(n+\zeta)},\ i = 1, \ldots, 2n. \tag{6c}$$

Here β is used to incorporate prior knowledge of the distribution of state X_t (with $\beta = 2$ used for Gaussian distributions [22]).

2. Update and filter: A new set of sigma points, $\mathcal{X}_t^-$, are formed from the predicted mean and covariance according to Eq (3) using $\mathbf{m}_t^-$ and $\mathbf{P}_t^-$ in place of $\mathbf{m}_{t-1}$ and $\mathbf{P}_{t-1}$. These sigma points are then propagated through the measurement model Eq (1b)

$$\hat{\mathcal{Y}}_t^{(i)} = h(\mathcal{X}_t^{-(i)}),\ i = 0, ..., 2n, \tag{7}$$

[1]Such as Scardua and da Cruz, "Complete offline tuning of the unscented Kalman filter," Automatica, 80:54–61, 2017

and combined to form

$$\mu_t = \sum_{i=0}^{2n} W_i^{(m)} \hat{\mathcal{Y}}_t^{(i)}, \tag{8a}$$

$$S_t = \sum_{i=0}^{2n} W_i^{(c)} (\hat{\mathcal{Y}}_t^{(i)} - \mu_k)(\hat{\mathcal{Y}}_t^{(i)} - \mu_k)^T + \mathbf{R}_t, \tag{8b}$$

$$C_t = \sum_{i=0}^{2n} W_i^{(c)} (\mathcal{X}_t^{-(i)} - \mathbf{m}^-)(\hat{\mathcal{Y}}_t^{(i)} - \mu_t)^T, \tag{8c}$$

$$K_t = C_t S_t^{-1}, \tag{8d}$$

where $\mathbf{R}_t$ is the covariance matrix of the measurement noise v_t in Eq (1b). Finally, these are used to produce the filtered estimates of the mean and covariance of the process at time t using the data up to time t through

$$\mathbf{m}_t = \mathbf{m}_t^- + K_t \left[Y_t - \mu_t \right], \quad \mathbf{P}_t = \mathbf{P}_t^- - K_t S_t {K_t}^T. \tag{9a}$$

Unscented Rauch-Tung-Striebel Smoother (URTSS)

The URTSS iterates backwards from $t = N, N-1, ..., 0$, beginning with the final results of the UKF, $\mathbf{m}_T^s = \mathbf{m}_T$ and $\mathbf{P}_T^s = \mathbf{P}_T$, as follows.

1. Prediction and update: Form the sigma points $\mathcal{X}_t$ from Eq (3) using $\mathbf{m}_t$ and $\mathbf{P}_t$, propagate them through the motion model

$$\hat{\mathcal{X}}_{t+1}^{(i)} = f(\mathcal{X}_t^{(i)}, w_t, \theta), \; i = 0, 1, ..., 2n, \tag{10}$$

and then combine the predictions by using

$$\mathbf{m}_{t+1}^- = \sum_{i=0}^{2n} W_i^{(m)} \hat{\mathcal{X}}_{t+1}^{(i)}, \quad \mathbf{P}_{t+1}^- = \sum_{i=0}^{2n} W_i^{(c)} (\hat{\mathcal{X}}_{t+1}^{(i)} - \mathbf{m}_{t+1}^-)(\hat{\mathcal{X}}_{t+1}^{(i)} - \mathbf{m}_{t+1}^-)^T + Q_t, \tag{11a}$$

$$D_{t+1} = \sum_{i=0}^{2n} W_i^{(c)} (\mathcal{X}_t^{(i)} - \mathbf{m}_t)(\hat{\mathcal{X}}_{t+1}^{(i)} - \mathbf{m}_{k+1}^-)^T, \tag{11b}$$

where the weights are given in Eq (6).

2. Produce the smoothed estimate: The mean and covariance of the smoothed

Gaussian density at time t are calculated from

$$\mathcal{G}_t = D_{k+1}\left[P^-_{t+1|N}\right]^{-1}, \tag{12a}$$

$$\mathbf{m}^s_{t|N} = \mathbf{m}_t + \mathcal{G}_t(m^s_{t+1|N} - \mathbf{m}^-_{t+1}), \quad \mathbf{P}^s_{t|N} = \mathbf{P}_t - \mathcal{G}_t(\mathbf{P}^s_{t+1|N} - \mathbf{P}^-_{t+1})\mathcal{G}^T_t. \tag{12b}$$

E-step via UKF and URTSS

From the results of the UKF and URTSS, the (approximate) posterior densities needed for the EM algorithm are

$$p(X_t|Y_{1:N}) \sim \mathcal{N}(\mathbf{m}^s_{t|N}, \mathbf{P}^s_{t|N}), \tag{13a}$$

$$p(X_t, X_{t-1}|Y_{1:N}) \sim \mathcal{N}\left(\begin{bmatrix}\mathbf{m}^s_{t|N}\\ \mathbf{m}^s_{t-1|N}\end{bmatrix}, \begin{bmatrix}\mathbf{P}^s_{t|N} & \mathbf{P}^s_{t|N}\mathcal{G}^T_{t-1}\\ \mathcal{G}_{t-1}\mathbf{P}^s_{t|N} & \mathbf{P}^s_{t-1|N}\end{bmatrix}\right). \tag{13b}$$

This gives the approximation of the $\mathcal{Q}$ function to be

$$\begin{aligned}\mathcal{Q}(\theta, \hat{\theta}^{(i)}) &\approx -\frac{1}{2}\log(2\pi\mathbf{P}_0) - \frac{1}{2}\log(2\pi\mathbf{Q}) - \frac{1}{2}\log(2\pi\mathbf{R})\\ &-\frac{1}{2}tr\left\{\mathbf{P}_0^{-1}\left[\mathbf{P}^s_{0|N} + (\mathbf{m}^s_{0|N} - \mathbf{m}_0)(\mathbf{m}^s_{0|N} - \mathbf{m}_0)^T\right]\right\}\\ &-\frac{1}{2}\sum_{t=1}^{N} tr\left\{\mathbf{Q}^{-1}\mathbb{E}\left[(x_t - f(x_{t-1}))(x_t - f(x_{t-1}))^T|Y_N\right]\right\}\\ &-\frac{1}{2}\sum_{t=1}^{N} tr\left\{\mathbf{R}^{-1}\mathbb{E}\left[(y_t - h(x_t))(y_t - h(x_t))^T|Y_N\right]\right\},\end{aligned} \tag{14}$$

where $\mathbf{Q}, \mathbf{R}$ are the covariance matrices for the motion model and observation model respectively, $\mathbf{P}_0, \mathbf{m}_0$ are initial estimate of the motion covariance and mean state, $\mathbf{P}^s_{0|N}, \mathbf{m}^s_{0|N}$ are smoothed estimates of the motion covariance and mean state at the initial time, and tr denotes the trace operation.

M-step for Parameter Estimation

The application of the maximization step under the U-EM scheme yields an

analytical expression for the estimate of D_x at the eth EM iteration, given by

$$\hat{D}_{x,e} = \frac{1}{2N\Delta t}\cdot\Big[\sum_{t=1}^{N}(\hat{x}^2_{t|N,e} + P_{t|N,e}) + \sum_{t=1}^{N}(\hat{x}^2_{t-1|N,e} + P^2_{t-1|N,e}) - 2\sum_{t=1}^{N}(\hat{x}_{t|N,e}\cdot\hat{x}_{t-1|N,e} + P_{t,t-1|N,e})\Big]. \quad (15)$$

The analytical expression for $\hat{D}_{y,e}$ is analogous to Eq (15).
