# Supplementary material for: Expectation maximization based framework for joint localization and parameter estimation in single particle tracking from segmented images
Source: PLoS One. 2021 May 21;16(5):e0243115. doi: 10.1371/journal.pone.0243115 (PMC8139521; doi:10.1371/journal.pone.0243115)
Supplement: S2 Text — (PDF) [file pone.0243115.s002.pdf]

S2 Text. Detailed description of SMC-EM.

Consider a generic dynamic state space system depending on a parameter θ

$$X_{t+1} = f_t(X_t, w_t, \theta), \tag{1a}$$

$$Y_t = h_t(X_t, v_t, \theta). \tag{1b}$$

The SMC-EM can use arbitrary number of randomly generated samples (often referred to as *particles*) to approximate the posterior needed by the EM algorithm. Under the SMC-EM scheme, a particle filter (PF) provides the filtered state estimates and importance weights. These are then passed to a particle smoother (PS) that works backwards to produce the smoothed distribution. These steps are described below.

Particle Filter (PF)

In this work, for simplicity we use a basic sequential importance resampling PF. Alternative PFs with different numerical properties could also be used[1].

1. Randomly generate M initial particles at time $t = 0$ by drawing from the initial distribution $P_\theta(x_0)$,

$$x_0^i \sim P_\theta(x_0), \quad i = 1, ..., M. \tag{2}$$

2. Propagate these M particles through the motion model Eq (1a),

$$\tilde{x}_t^i = f_{t-1}(\tilde{x}_{t-1}, w_{t-1}, \theta), \quad i = 1, 2, ..., M. \tag{3}$$

3. Compute the importance weights,

$$w_t^i \triangleq \tilde{w}_t^i = \frac{P_\theta(Y_t|\tilde{x}_t^i)}{\sum_{j=1}^M P_\theta(Y_t|\tilde{x}_t^j)}, \quad i, j = 1, ..., M. \tag{4}$$

where Y_t is the observed measurement at time t and $P_\theta(Y_t|\tilde{x}_t^i)$ is determined by the measurement model in Eq (1b).

[1] see, e.g., Doucet A, Johansen AM. A tutorial on particle filtering and smoothing: Fifteen years later. In: Crisan D, Rozovskii B, editors. The Oxford Handbook of Nonlinear Filtering. Oxford: Oxford University Press; 2011. p. 656-704.

4. Resample M new particles x_t^j from the discrete distribution,

$$p(x_t^j = \tilde{x}_t^i) = w_t^i,\ j = 1, \ldots, M. \tag{5}$$

5. Increment $t \leftarrow t+1$ and iterate from step 2 until $t = N$.

Particle Smoother (PS)

The PS used in this work is also known as forward-filtering backward smoothing. As with the PF, other particle smoothing schemes can be selected.

1. Initialize the importance weights w_t^i at $t = N$ as $w_{N|N}^i = w_t^i$, $i = 1, ..., M$.

2. Decrement $t \leftarrow t-1$ and calculate the smoothed weights $w_{t|N}^i$ by backward computation,

$$w_{t|N}^i = \sum_{j=1}^{M} w_{t+1|N}^j \frac{w_t^i P_\theta(\tilde{x}_{t+1}^j|\tilde{x}_t^i)}{\sum_{l=1}^{M} w_t^l P_\theta(\tilde{x}_{t+1}^j|\tilde{x}_t^l)}, \tag{6}$$

where $P_\theta(\tilde{x}_{t+1}^j|\tilde{x}_t^i)$ is determined by the motion model in Eq (1a).

3. Iterate from Step 2 until $t = 0$.

E-step via PF and PS

Using the results of the PF and PS, the $\mathcal{Q}$ function can be approximated by

$$\mathcal{Q}\left(\theta, \theta^{(e)}\right) = I_1(\theta, \theta^{(e)}) + I_2(\theta, \theta^{(e)}) + I_3(\theta, \theta^{(e)}) \tag{7}$$

where

$$I_1(\theta, \theta^{(e)}) \approx \sum_{i=1}^{M} w_{1|N}^i log\ P_\theta(\tilde{x}_1^i), \tag{8a}$$

$$I_2(\theta, \theta^{(e)}) \approx \sum_{t=1}^{N-1} \sum_{i=1}^{M} \sum_{j=1}^{M} w_{t|N}^{ij} log P_\theta(\tilde{x}_{t+1}^j|\tilde{x}_t^i), \tag{8b}$$

$$I_3(\theta, \theta^{(e)}) \approx \sum_{t=1}^{N} \sum_{i=1}^{M} w_{t|N}^i log P_\theta(y_t|\tilde{x}_t^i). \tag{8c}$$

where $w_{t|N}^{ij}$ are given by

$$w_{t|N}^{ij} = \frac{w_t^i w_{t+1|N}^j P_\theta(\tilde{x}_{t+1}^j|\tilde{x}_t^i)}{\sum_{l=1}^{M} w_t^l P_\theta(\tilde{x}_{t+1}^j|\tilde{x}_t^l)}. \tag{9}$$

One benefit of SMC-EM over U-EM is its ability to represent arbitrary posterior distributions rather than approximating them as Gaussians.

M-step for Parameter Estimation

The application of the maximization step under the SMC-EM scheme yields an analytical expression for the estimate of D_x at the eth EM iteration, given by

$$\hat{D}_{x,e} = \frac{1}{2N\Delta t}(D^a_{x,e} + D^b_{x,e}), \tag{10}$$

with

$$D^a_{x,e} \triangleq \sum_{i=1}^{M} w^i_{1|N,e}(x^i_{1|N,e})^2, \quad D^b_{x,e} \triangleq \sum_{k=1}^{N-1}\sum_{i=1}^{M}\sum_{j=1}^{M} w^{ij}_{k|N,e}(x^j_{k+1|N,e} - x^i_{k|N,e})^2.$$

where $x^i_{t|N,e}$ denotes the smoothed state for the ith sampling particle at time t, and M is the number of samples used in the PF/PS. The analytical expression for estimated D_y is analogous to Eq (10).
