# Supplementary material for: Expectation maximization based framework for joint localization and parameter estimation in single particle tracking from segmented images
Source: PLoS One. 2021 May 21;16(5):e0243115. doi: 10.1371/journal.pone.0243115 (PMC8139521; doi:10.1371/journal.pone.0243115)
Supplement: S3 Text — (PDF) [file pone.0243115.s003.pdf]

S3 Text. Gain and covariance simulation based on statistical data.

Our specific model for an sCMOS camera is based on that of Ref. [38] of the main paper. That work used a Hamamatsu ORCA Flash 4.0 camera and provided experimental data on the pixel-by-pixel variance and gain. We fit the variance data with the model

$$\log_{10} \text{Occurance} = a \cdot \exp\left(-b \cdot \text{variance}\right), \tag{1}$$

finding the best fit parameters to be $a = 4.734, b = -0.001799$. Similarly, we fit the gain data to the model

$$\log_{10} \text{Occurance} = a_1 \cdot \exp -(\frac{g - b_1}{c_1})^2 + a_2 \cdot \exp -(\frac{g - b_2}{c_2})^2, \tag{2}$$

leading to the values

$$a_1 = 2.215, b_1 = 2.19, c_1 = 0.07661, a_2 = 2.68, b_2 = 2.249, c_2 = 0.216$$

.

In the figure below we show maps of the pixel-dependent variance and gain for an 512×512 image respectively as well as the curve fitting results.

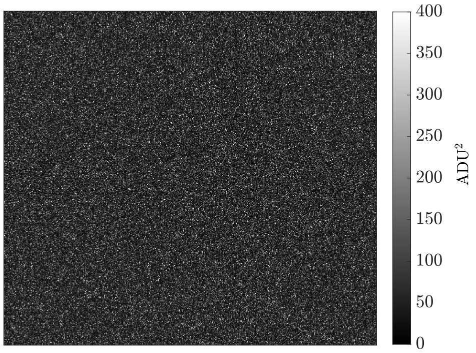

(a) Simulated variance map.

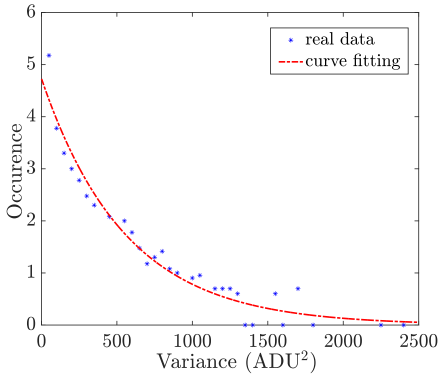

(b) Variance curve-fitting.

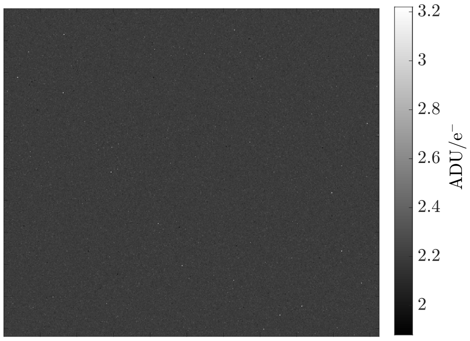

(c) Simulated gain map.

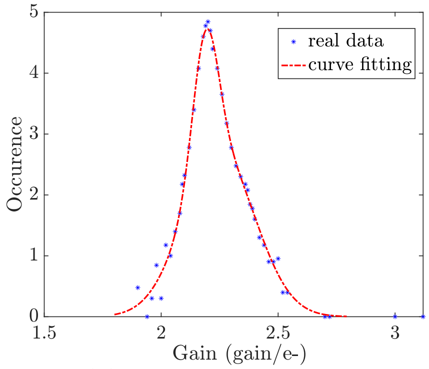

(d) Gain curve-fitting.

Simulated maps and histograms of the pixel-dependent readout noise.
