# Supplementary material for: Expectation maximization based framework for joint localization and parameter estimation in single particle tracking from segmented images
Source: PLoS One. 2021 May 21;16(5):e0243115. doi: 10.1371/journal.pone.0243115 (PMC8139521; doi:10.1371/journal.pone.0243115)
Supplement: S4 Text — (PDF) [file pone.0243115.s004.pdf]

S4 Text. Probability density function of measurements considering pixel dependent readout noise.

From Ref [38] of the main text, the probability density function (PDF) of the measured ADU (analog-to-digital units) counts $C_{p,t}$ in the pth pixel at time t is a combination of the Poisson photon noise and the Gaussian read-out noise and can be expressed as

$$P(C_{p,t}) = A \sum_{q=0}^{\infty} \frac{1}{q!} \exp^{-\mu_{p,t}} \mu_{p,t}^{q} \frac{1}{\sqrt{2\pi \mathrm{Var}_{p,t}}} \exp^{-\frac{\left[\frac{C_{p,t}-O_{p,t}}{g_{p,t}}-q\right]^2}{2\mathrm{Var}_{p,t}/g_{p,t}^2}}, \tag{1}$$

where A is a normalizing constant, $\mu_{p,t}$ is the number of expected photoelectrons arising from signals and background noise, $g_{p,t}$ is the amplification gain, $O_{p,t}$ and $\mathrm{Var}_{p,t}$ are the offset and variance of the readout noise in pixel p at time t, respectively.

From this distribution, the measured photon counts $I_{p,t}$ for the pth pixel at time t is given by

$$\frac{I_{p,t}}{QE} \triangleq \frac{C_{p,t} - O_{p,t}}{g_{p,t}} \approx \mathrm{Poiss}(\mu_{p,t}) + \epsilon_{p,t}, \tag{2a}$$

$$\epsilon_{p,t} \sim \mathcal{N}(0, \sigma_{p,t}^2), \tag{2b}$$

$$\sigma_{p,t}^2 = \frac{\mathrm{Var}_{p,t}}{g_{p,t}^2}, \tag{2c}$$

where QE is the quantum efficiency of the camera pixels. In this work, for simplicity, we ignore the offsest as it is a constant shift, and set $QE = 1$ for simplicity. Therefore, in our case, the measured photon counts $I_{p,t}$ for the pth pixel at time t can be expressed as

$$I_{p,t} \sim \mathrm{Poiss}(\mu_{p,t}) + \epsilon_{p,t}, \tag{3}$$

with

$$\mu_{p,t} = \lambda_{p,t} + N_{bgd}, \tag{4}$$

where $\lambda_{p,t}$ is the expected photon intensity arising from signals, following the double integral form as Eq (14) of the main paper, and N_{bgd} is the photon counts arising from background noise.

The PDF of the measured photon counts is expressed as

$$P(I_{p,t}) = \sum_{q=0}^{\infty} \frac{1}{q!} \exp^{-(\lambda_{p,t}+N_{bgd})} (\lambda_{p,t} + N_{bgd})^q \frac{1}{\sqrt{2\pi\sigma_{p,t}^2}} \exp^{-\frac{(I_{p,t}-q)^2}{2\sigma_{p,t}^2}}, \tag{5}$$

where all values of $\mathrm{Var}_{p,t}$ and $g_{p,t}$ that define $\sigma_{p,t}$ depend on the type of camera sensors used for imaging. If the readout noise is neglected, we set $\epsilon_{p,t} = 0$; if a CCD/EMCDD camera sensor is modeled, we set $\sigma_{p,t}$ as a constant; if a CMOS/sCMOS is modeled, the value depends on the pixel.
