# Supplementary material for: Expectation maximization based framework for joint localization and parameter estimation in single particle tracking from segmented images
Source: PLoS One. 2021 May 21;16(5):e0243115. doi: 10.1371/journal.pone.0243115 (PMC8139521; doi:10.1371/journal.pone.0243115)
Supplement: S5 Text — (PDF) [file pone.0243115.s005.pdf]

S5 Text. Analytical approximation of $\mathbf{MLE_{sCMOS}}$

Following Ref [38] of the main paper, the probability distribution function for a pixel value with both shot noise and read-out noise can be approximated by

$$
\begin{aligned}
&P_{\text{sCMOS}}(z = I_{p,t} + \sigma_{p,t}^2 | \lambda_{p,t}(\theta_{xy,t}), N_{bgd}, g_{p,t}, \text{Var}_{p,t}) \\
&\quad = \frac{e^{-(\lambda_{p,t}+N_{bgd}+\sigma_{p,t}^2)}(\lambda_{p,t} + N_{bgd} + \sigma_{p,t}^2)^z}{\Gamma(z+1)},
\end{aligned} \tag{1}
$$

where $\Gamma(\cdot)$ is the standard Gamma function. The $\text{MLE}_{\text{sCMOS}}$ for localization at time t can be expressed as

$$
\hat{\theta}_{xy,t} = \underset{\theta_{xy,t}}{\arg\min} \left\{ -\ln \left[\prod_{p=1}^{25} P_{\text{sCMOS}}(z = I_{p,t} + \sigma_{p,t}^2 | \lambda_{p,t}(\theta_{xy,t}), N_{bgd}, g_{p,t}, \text{Var}_{p,t}) \right] \right\}, \tag{2}
$$

where $\hat{\theta}_{xy,t}$ is the maximum log-likelihood estimation of localization at time t.

Using the form of the PSF for $\lambda_{p,t}$ given in the main paper, this MLE can be expressed as

$$
\hat{\theta}_{xy,t} = \underset{\theta_{xy,t}}{\arg\min} \sum_{p=1}^{25} \left[(\lambda_{p,t} + N_{bgd} + \sigma_{p,t}^2) - z \cdot \ln(\lambda_{p,t} + N_{bgd} + \sigma_{p,t}^2) + \ln \Gamma(z+1) \right], \tag{3}
$$

where $z = I_{p,t} + \sigma_{p,t}^2$.
