# Supplementary material for: Expectation maximization based framework for joint localization and parameter estimation in single particle tracking from segmented images
Source: PLoS One. 2021 May 21;16(5):e0243115. doi: 10.1371/journal.pone.0243115 (PMC8139521; doi:10.1371/journal.pone.0243115)
Supplement: S1 Video — (PDF) [file pone.0243115.s006.pdf]

S1 Video. A typical video showing the relationship among trajectory, observation, and properties of readout noise brought by sCMOS.

In this video, the top left figure shows the ground truth trajectory of the diffusing particle, indicating both the current location of the particle (green +) and the current area of the 5 pixel $\times$ 5 pixel image that is input to the algorithm. The top right figure is that image, the bottom left is the variance of each pixel in that same region, and the bottom right is the gain of each pixel in that region.
