# Supplementary figures and images for: Expectation maximization based framework for joint localization and parameter estimation in single particle tracking from segmented images
Source: PLoS One. 2021 May 21;16(5):e0243115. doi: 10.1371/journal.pone.0243115 (PMC8139521; doi:10.1371/journal.pone.0243115)

S1 Fig. Computation time record for different SPT algorithms.

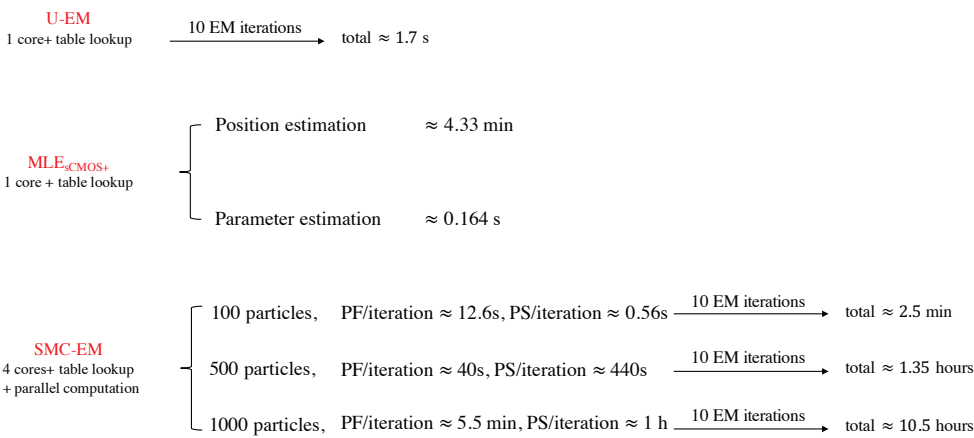

Supplement: S1 Fig — (PDF) [file pone.0243115.s007.pdf]
