# Supplementary material for: Expectation maximization based framework for joint localization and parameter estimation in single particle tracking from segmented images
Source: PLoS One. 2021 May 21;16(5):e0243115. doi: 10.1371/journal.pone.0243115 (PMC8139521; doi:10.1371/journal.pone.0243115)
Supplement: Attachment — Submitted filename: ResponseToReviewers_final.docx [file pone.0243115.s008.docx]

|  | Sean B. Andersson,  Professor  Mechanical Engineering and the Division of  Systems Engineering  Boston University, Boston, MA 02215. USA  617.353.4949  sanderss@bu.edu |
| --- | --- |

To the Editorial Staff,

We would like to thank the Editorial Staff and the Reviewers for their careful attention to our submission PONE-D-20-35561, Expectation Maximization based framework for joint localization and parameter estimation in single particle tracking from segmented images. We have done our best to respond to all reviewer concerns.

In addition, we have addressed the additional requirements noted by the Academic Editor. Specifically:

1. *Please ensure that your manuscript meets PLOS ONE’s style requirements.*

We have re-formatted the captions of all figures and tables and checked the other formatting requirements to ensure they meet PLOS ONE requirements. This includes reformatting the manuscript to double spaced.

2. *Upon re-submitting your revised manuscript, please upload your study’s minimal underlying data set as either Supporting Information files or to a stable, public repository and include the relevant URLs, DOIs, or accession numbers within your revised cover letter.*

We submitted our dataset to the DRYAD data repository (<https://datadryad.org/>). DOI: 10.5061/dryad.9w0vt4bf5. Note that the DOI is not yet active, pending the outcome of the manuscript. The data can currently be accessed at the link:

<https://datadryad.org/stash/share/gS8Q1F-4IgxGB5QO-ibFkVO62rx24-mLT29ME8wqI3s>

3. *Please include captions for your Supporting Information files at the end of your manuscript and update any in-text citations to match accordingly.*

We have made this correction.

Please note that while checking all of our figures, we uncovered a mismatch in the data used in Figure 4 (which shows the time traces and the corresponding 2-D trajectory). We have corrected this in the revision. (This error did not affect any of the calculations and was merely one of presentation.)

Below we detail our response to each reviewer comment. All changes to the manuscript are shown in blue text in the marked-up copy of the manuscript. We also fixed any small typographic errors that we found; we did not mark these up in blue. Please note that in the marked up version we also included all the supplemental material (with blue text) for easy reference of the reviewers.

**REVIEWER 1**

*This paper proposes a method to jointly estimate the location of a single particle which diffuses on a media with unknown diffusion parameters. Assuming previously segmented images, the method iteratively combines a classical 2D position tracker with an ME estimator for the diffusion coefficients. Since motion depends on these coefficients, it is reasonable to estimate localization and parameters jointly, which is the role of the iterative loop. Both the particle size and the motions to estimate are in the order of the nm and thus the paper focuses greatly on the effect that the different sources of noise have on final estimate. The methods are tested in simulation only.*

*The paper is well written and for the most part it follows easily.*

We thank the reviewer for their careful read and constructive comments on our work.

*However, I have been surprised by the organization of the presented material. This is possibly due to the editor’s constraints, but I do not see why the methods are coming as separate material labelled “supplemental”. I understand that if an article is about studying a particular phenomenon, particularly in the Natural Sciences, then the methods can be assumed supplemental (in case these methods are well-known or at least published elsewhere). But the present paper is about the methods themselves, since all the analyzed data is simulated and therefore do not reflect any real phenomenon (only realistic). In my opinion, S1 and S2 at least should go in the main body, and they should be the basis upon which to base our evaluation.*

We agree with the reviewer that our paper is about the methods but feel that its primary contribution is the quantitative comparison made between the different algorithms considered and in the range of imaging conditions and types of cameras considered. It is very common in the biophysical community to find all details of the algorithms relegated to the Supplemental Materials, focusing the main body of the paper on the results. (We recognize that it is absolutely not common in many other domains such as, for example, the robotics literature.) We have done our best to ensure the main body contains sufficient descriptions of the algorithms for the reader to understand the comparisons that follow but have chosen to keep the detailed descriptions in the Supplemental Materials, adding a few additional comments into the main sections to better describe the algorithms and their limitations.

*The key contributions can be divided in two parts. First, there are the methods. Second, there is a detailed benchmark of these methods compared with some predecessors as they have been introduced in the state of the art sections.

The paragraph above about the organization of the paper is pertinent for the first part, that is, the presentation of the methods. Although the methods are correct and seem to improve on the state of the art. Here I assume the state of the art is that of the particular discipline of particle tracking in biological macromollecules, for which I am not competent. If this state of the art is correct, then I must say that the community needs to make an effort because there is a lot of room for improvement (see a few paragraphs below).*

We completely agree that there is room for improvement and believe that this particular submission is a step in that direction!

Concerning this paper, I think the presented methods fall short in some aspects, discussed below.

*First, the model parameters to estimate are very simplistic. Having a medium with anisotropic diffusion, the pair Dx, Dy is only pertinent if one aligns perfectly the material with the reference frame. I would have liked to see at least a third parameter estimated, that is the relative orientation of the medium with respect to the reference frame, or equivalently, a covariance matrix representing this diffusion as e.g. D = [Dxx, Dxy ; Dxy, Dyy] (or similar). In case of aligned frames we’d have Dxy=0, Dxx=Dx^2, Dyy=Dy^2.*

We agree that assuming two independent axes is a strong assumption and valid only if the medium is aligned to the reference frame. In fact, in most of the literature on estimation of diffusive motion it is assumed the diffusion is isotropic. Both the standard Mean Square Displacement algorithm (still the standard in the biophysical community) and the Maximum Likelihood algorithm used here (and derived in ref. 16) assume either isotropic diffusion or independent axes. In order to compare our results against them, we assumed the independent axes model. However, accounting for the cross terms is straightforward under the EM scheme. We have added some brief text after the motion model in Eq. (12) highlighting the more general case.

*Second, the UKF has some design parameters that are worth exploring. In particular, one chooses \alpha in the range [0,1] (S1, paragraph after eq. 3) to decide how the control points are located around the mean to approximate the covariance. In the results section, the authors state that U-EM does not perform well in some cases due probably to a deficient sampling, which makes this paramter \alfa very pertinent. Since the methods are just presented quickly and fall in the supplemental material, they are poorly discussed. The reader is left with the impression that better results could be obtained, or otherwise that a proper discussion of the limits of the method (UKF in this case) is missing.*

It is true that there is more to the UKF than it may seem at first blush. In fact, as the reviewer likely knows, there are many variations on these types of filters, such as the cubature filter (see, e.g., Arasaratnam and Haykin, Cubature Kalman Filters, IEEE Trans. Automatic Control, 54(6):1254-1269, 2009) as well as methods for tuning these parameters (see, e.g. Scardua and da Cruz, ``Complete offline tuning of the unscented Kalman filter,'' Automatica, 80:54–61, 2017). In this work we chose to focus on a straightforward version of the UKF as a common choice of ``advanced’’ filter.

We have added a brief comment into the main text about the cubature filter variant, including the Arasaratnam and Haykin reference, as well as additional text into S1 (with the U-EM details) about tuning of the parameters (including the Scardua and da Cruz reference).

*Third, the estimation problem posed in this paper seems very simple. In the area of robotics, for example, we are used to estimate the location of a robot in space, together with many other parts of the system (location of objects in the environment, sensor calibration parameters, velocities and acceleration, time delays), all this in 3d (that is, with 6DoF). These works deal with nonlinear systems with hundreds or thousands of variables, while the method presented here only estimates four parameters, and only for a single particle. The estimation literature also contains works to jointly estimate the covariance of the perturbations to the system, which in the present paper are the diffusion parameters. I would recommend the readers to have a look at the robotics literature on simultaneous localization and mappping (a term which reminds the title of the present paper), for example using EKF (Davison 07), UKF (Sotoodeh Bahraini 15), PF (Thrun 04), (Campi 08, this one used not in robotics but in magnetoencephalography), or factor graphs (Kaess 11).*

We thank the reviewer for these suggestions. In general, while we are at least partially aware of the SLAM literature, we have typically thought of these two problems in very different terms. The robotics setting does typically estimate a full 6-DOF pose, and sometimes even the 12-D setting with velocities included. By contrast, at the nanometer-scale of macromolecular biophysics, inertia plays no role (so that kinematic models are accurate representations of the dynamics) and orientation is almost never considered (since the measurements contain no information about the physical details of the particle being tracked and the motion does not depend on the particle orientation, at least not strongly). As a result, we have been pulling primarily from the fields of signal processing and optimal estimation.

Of course, just because this has been our approach to date does not mean that we should be ignoring the robotics literature! Once again, we are grateful for the suggestions and will take a careful look at the references provided.

*For the second part of the contributions, that is the benchmarking of results, I have to say that I found it very interesting, for it is my first contact with experiments in the nano-scale. Although I cannot judge it, I found the discussion on the error models interesting. I have two remarks. The first one would be that of the tuning of the UKF, as said above, which if explored could have changed the panorama of the results presented, perhaps only slightly but anyway worth exploring.*

We are glad you found the benchmarking results interesting and appreciate the suggestion of considering the tuning of the UKF. As noted, we explored parameter tuning and found that in general selecting alpha = 1 yielded good performance across the range of viable diffusion coefficients (and tuning did not appreciably extend that range). To better highlight this, we added the text in the supplemental material S1 where the parameter alpha appears.

*The second remark concerns the observable estimation bias clearly visible in Figs. 5 and 6, where the true values seem to be always at the top of the third quartile, sometimes slightly outside of it. Again, being an article about the estimation methods themselves, a commentary here I feel is absolutely necessary. Often, such biases appear due to nonlinearity, but can also appear due to non-Gaussian noise. Both causes are taken care of by UKF and PF, and therefore one would ideally expect these biases to be absent.*

This bias is indeed something we typically see in our estimation results and we agree that we should have commented on them in the article. The bias appears to be driven by two elements. The first is the relatively short length of the data (100 time steps). The GF-MLE and the EM based schemes all utilize maximum likelihood estimation. Since the MLE is known to be consistent, we in general expect unbiased results. However, consistent only means that the MLE approaches the true value in the limit of large data. The second is, as the reviewer notes, the nonlinearities in the system. In fact, a close look at the results shows that the bias reduces (slowly!) as the number of particles in the PF increases from 100 to 1000, supporting the hypothesis that the underlying nonlinearities play a role. The UKF does handle the nonlinearities but is only accurate to second order in representing the underlying distributions that are used in the EM step. We have added text in the main paper following the introduction of the results in Fig. 6 to better note and describe the bias.

*The rest of my comments are minor (typos and the like, small questions and improvements for clarity):*

*1. Fix this: “Q(θ(e+1), θ(e)) ≥ Q θ(e), θ(e)*

*ensures the EM algorithm converges to a local *minimum of the likelihood function.” —> local maximum/optimum of the likelihood function*

Fixed.

*2. In S1, (13,14,15), is T=N? Is this a typo?*

We apologize for any confusion here. N is the data length (number of images), while lower case ‘t’ is the time step index. Any instance of ‘T’ indicates the transpose operation. We added a brief comment at the start of S1 (after the model in (1)) to remind the reader of the role of N.

*3. PSF in (13), maybe center the distribution at (x0, y0) as was done in (1).*

Thank you for the suggestion. As you surmised, the formulation in (13) centers the particle at the origin while (1) centers the intensity pattern on the particle. We have adjusted the definition of the PSF in (13) to match that of (1) to avoid confusion (and adjusted the expression in (14) accordingly).

*4. Fig 5 (right) use equal X and Y scales*

Done. Note that in making this adjustment, we also noticed a typo in the right subfigure of Fig. 5(d) where the vertical axis was mistakenly labeled “D_x” instead of “D_y”. We fixed this as well.

*5. Fig 7: use better a logscale for G, e.g. 1,2,5,10,20,50,100 or finer, but not a two-piecewise linear scale as shown.*

Done.

*6. Fig 7: label the color scale (color corresponds to…) and a percentage is not [0,1] but [0,100]*

We have adjusted the color bar to be percentage instead of fraction (to match the text), added a label to the color bar, and adjusted the figure caption to describe the color code.

*7. End of page 10. Add a dot after “G=3” in “at an intensity of G = 3 With an SBR of 10”.*

Done.

*8. Fig 9 I’d prefer (nm) and not (um) in the Y scales, to relate them to previous figures in the text which are in nm.*

Done.

*9. Fig 9 caption: add a dot after “(dashed line)”*

Done.

*10. Results case 3: written Fig *9 —> must be Fig *10 !!*

Thank you for catching this (and other) typos. Fixed.

*11. Does keeping Dt/dt = constant make sense? At lower shutter time dt, there might be no image quality at all?*

You are correct that as the shutter time goes to zero, so does the signal level (assuming the peak intensity is held constant). The challenge for this comparison is that performance depends on a variety of parameters. Localization in particular depends in large part on the number of photons received; if one ignores the camera read-out noise and motion blur, it is a shot-noise process and thus precision scales (at least in the limit of large signal) as 1/sqrt(N) where N is the number of photons. Including motion blur means that this will decay further as the integration time is increased. Our goal with the expressions for a maximum D was to provide some guidance beyond the specific results where the value depends on the choices made. Upon reflection, it is not clear that these expressions provide any such guidance. Thus, in the revision we have omitted them and instead inserted a brief description of (at least some of) the factors that influence the threshold.

*12. Many other places missing a dot at end of sentence. e.g. “low (G = 10) and one high (G = 100) A typical image frame” in page 12. Please check thoroughly.*

Thank you for nothing these. We have gone through the entire document and done our best to catch these (and similar) minor typos.

*13. Discussion and *Ccnclusion —> Conclusion*

Fixed.

Video: not clear what are the axes of the pixelated images. Do they cover the whole area of the figure top-left? If yes, this is weird since in top-right the particle seems to be always in the center. If not, then please specify.

We apologize for the confusion. The figure on the top-left of the movie denotes the global position with a size selected large enough to show the entire trajectory. The other figures are local 5 x 5 sub-areas centered on the current location of the particle and represent the images that are the input to our algorithm (top right) and the variance and gain maps that define the sCMOS camera observation model on those same 5 x 5 pixels (bottom two). To clarify the movie, we have made several changes, including modifying the titles of each of the sub-images, adding a green + symbol in the upper left plot to indicate the location of the particle at the current time step, and a red square in that same figure to indicate the area covered by the 5 x 5 pixel array. We have also added a description of the video into the supplemental material.

**REVIEWER 2**

*In this article, the authors highlight simultaneous identification and diffusion coefficient determination for the single particle tracking, with an emphasis on 2-D diffusive systems. Two different methods were introduced: Sequential Monte Carlo-Expectation Maximization (SMC-EM) and Unscented Kalman Filter (UKF) . They envisioned that EM based methods outperform the standard techniques, especially at low signal levels. Their algorithm operates at low and high diffusion rate, at low and high signal level and in two different cameras with different noise sources. They have thoroughly discussed the performances of the algorithms under various conditions. The authors also attempted to extend their algorithm to 3-D systems. This reviewer found this manuscript interesting and would recommend its acceptance after some minor revision.*

We thank the reviewer for their careful read and constructive comments on our work.

*1. The authors attempted to overcome the coupling problem for simultaneous localization and parameter estimation. To get success, they initially developed Sequential Monte Carlo-Expectation Maximization method for nonlinear system tracking which they published in 2015 (ref. 20). Due to the computational complexity the authors again modified the above method and address the Unscented Kalman filter (UKF) and an Unscented Rauch-Tung-Striebel smoother (URTSS). They also published this work in 2019 (ref. 21). The only new approach here they compare their method with standard method (e.g., GF-MSD and GF-MLE), range of signal to noise ratio, two different camera methods with different noise sources, and low and high diffusion rate. It would be great if the authors could emphasize the novelty of the current work and better distinguish it from the previous ones.*

We agree with the reviewer that the contribution was not clearly stated. We have added a new paragraph to the end of the introduction, describing the contribution as (a) extending our existing algorithms to include the sCMOS camera model and (b) performing a detailed, quantitative comparison to standard methods used in the community. We also slightly modified the Conclusions to highlight the contributions.

2. They separately defined the method for slow and fast particle diffusion. If the rate of particle diffusion in a sequence of images can differ with time, how can SPT be analyzed or is it accurately possible by these methods?

Thank you for your question. First, it is important to note that the method in this paper is, as you note, for fixed diffusion coefficient parameters. The algorithms, however, do not depend on whether that parameter is slow or fast, though their performance does. Second, analyzing trajectories with time-varying parameters using our EM-based methods is a topic of ongoing research; our group has just published a paper in Molecules on this very topic. This citation was added to the introduction.

3. Some minor edits:
- line 7: ‘been’ written double times

Fixed.

- Line 383: Spelling mistake ‘conclusion’

Fixed.

Sincerely,

Sean B. Andersson

Professor of Mechanical Engineering and Systems Engineering, Boston University
